# The Hyperpolarization-Activated Current Determines Synaptic Excitability, Calcium Activity and Specific Viability of Substantia Nigra Dopaminergic Neurons

**DOI:** 10.3389/fncel.2017.00187

**Published:** 2017-06-28

**Authors:** Carmen Carbone, Alessia Costa, Gustavo Provensi, Guido Mannaioni, Alessio Masi

**Affiliations:** ^1^Department of Neuroscience, Psychology, Drug Research and Child Health (NEUROFARBA), Section of Pharmacology and Toxicology, University of FlorenceFlorence, Italy; ^2^Toxicology Unit, Azienda Ospedaliero-Universitaria CareggiFlorence, Italy

**Keywords:** HCN channels, voltage-dependent calcium channels, patch clamp electrophysiology, excitotoxicity, Parkinson disease, differential vulnerability, mitochondria, dopaminergic neurons

## Abstract

Differential vulnerability between Substantia Nigra pars compacta (SNpc) and Ventral Tegmental Area (VTA) dopaminergic (DAergic) neurons is a hallmark of Parkinson’s disease (PD). Understanding the molecular bases of this key histopathological aspect would foster the development of much-needed disease-modifying therapies. Non-heterogeneous DAergic degeneration is present in both toxin-based and genetic animal models, suggesting that cellular specificity, rather than causing factors, constitutes the background for differential vulnerability. In this regard, we previously demonstrated that MPP+, a neurotoxin able to cause selective nigrostriatal degeneration in animal rodents and primates, inhibits the Hyperpolarization-activated current (Ih) in SNpc DAergic neurons and that pharmacological Ih antagonism causes potentiation of evoked Excitatory post-synaptic potentials (EPSPs). Of note, the magnitude of such potentiation is greater in the SNpc subfield, consistent with higher Ih density. In the present work, we show that Ih block-induced synaptic potentiation leads to the amplification of somatic calcium responses (SCRs) *in vitro*. This effect is specific for the SNpc subfield and largely mediated by L-Type calcium channels, as indicated by sensitivity to the CaV 1 blocker isradipine. Furthermore, Ih is downregulated by low intracellular ATP and determines the efficacy of GABAergic inhibition in SNpc DAergic neurons. Finally, we show that stereotaxic administration of Ih blockers causes SNpc-specific neurodegeneration and hemiparkinsonian motor phenotype in rats. During PD progression, Ih downregulation may result from mitochondrial dysfunction and, in concert with PD-related disinhibition of excitatory inputs, determine a SNpc-specific disease pathway.

## Introduction

Non-homogeneous degeneration within midbrain dopaminergic (DAergic) neurons is a histopathological hallmark of Parkinson’s disease (PD). Typically, DAergic neurons in the Substantia Nigra pars compacta (SNpc) are markedly more vulnerable than in the adjacent ventral tegmental area (VTA; Schapira, 2011; Brichta and Greengard, 2014). Numerous animal models, both toxin-based or transgenic, show non-uniform DAergic degeneration patterns, strongly suggesting that intrinsic cellular properties, rather than etiologic factors, underlie differential vulnerability between distinct subsets (Blesa and Przedborski, 2014). For therapeutic prospects, understanding the molecular bases of this key pathogenic feature would dramatically improve our chances to develop neuroprotective, disease-modifying treatments. Comparative SNpc-VTA gene expression studies have revealed extensively overlapping signatures between the two DAergic populations (Grimm et al., 2004; Greene et al., 2005), suggesting that quantitative, rather than qualitative differences in the expression or function of a limited number of genes subtend selective vulnerability. Over the last decade, it has been suggested that intrinsic electrophysiological properties of specific DAergic subsets, such as the differential expression or function of selected ion channels, provide a physiological substrate for differential vulnerability (Liss et al., 2005; Guzman et al., 2009, 2010; Surmeier et al., 2012; Dryanovski et al., 2013; Dragicevic et al., 2015). In this regard, we previously demonstrated that MPP+, a neurotoxin able to cause selective nigrostriatal degeneration in animal rodents and primates, inhibits the Hyperpolarization-activated current (Ih) in SNpc DAergic neurons (Masi et al., 2013) and that pharmacological Ih inhibition causes potentiation of evoked Excitatory post-synaptic potentials (EPSPs) preferentially in the SNpc (Masi et al., 2015). The role of Ih in nerve cell physiology has been extensively studied (He et al., 2014). Aberrant Ih function alters intrinsic and synaptic excitability in central and peripheral neurons, leading to the induction of epileptic states (DiFrancesco et al., 2011; DiFrancesco and DiFrancesco, 2015) or pathological pain signaling (Emery et al., 2012; Resta et al., 2016). The implications of Ih dysfunction in pathological states of the nervous system has prompted the quest for selective, subunit-specific modulators (Del Lungo et al., 2012; Novella Romanelli et al., 2016). In midbrain DAergic neurons, Ih has been the object of deep molecular and electrophysiological characterization (Mercuri et al., 1995; Seutin et al., 2001; Neuhoff et al., 2002; Lammel et al., 2008; Dufour et al., 2014; Krashia et al., 2017), but its implication in diseases of the DAergic system has remained largely unexplored. In the present work, we demonstrate that potentiation of synaptic excitability resulting from Ih inhibition leads to the amplification of somatic calcium responses (SCRs). The effect is specific for the SNpc subfield and largely, but not solely, mediated by L-Type calcium channels. We then show that Ih is downregulated in presence of low intracellular ATP and that Ih suppression reduces the inhibitory effect of GABAergic transmission, suggesting the existence of a mechanistic link between disruption of mitochondrial homeostasis and abnormal synaptic excitability in SNpc DAergic neurons. Finally, we tested the effect of Ih suppression *in vivo* and found that intracerebral stereotaxic injection of the selective blockers ivabradine or ZD7288 causes a pattern of DAergic degeneration strikingly resembling that seen in MitoPark mice and MPP+-treated mice, two distinct PD models characterized by mitochondrial failure and SNpc-specific DAergic degeneration (Ekstrand et al., 2007; Blesa and Przedborski, 2014). Overall, the present data support the hypothesis that Ih loss of function may represent a bona fide pathogenic mechanism in PD and thus a potential target for the future development of disease-modifying therapeutic interventions.

## Materials and Methods

### Midbrain Slice Preparation and Electrophysiological Recordings

All procedures required for *ex vivo* experiments were conducted in compliance with the Council Directive of the European Community (2010/63/EU), Decreto Legislativo Italiano 26 (13/03/2014) and approved by the Animal Care Committee of the Department of Neurofarba, University of Florence. Wistar rats of either sex at postnatal day 20–30, were anesthetized with isoflurane and decapitated. Midbrain horizontal slices (250 μm) were cut with a vibroslicer (VT1200S; Leica Microsystems Inc., IL, USA) in chilled artificial Cerebral Spinal Fluid (aCSF), composed of (in mM) 130 NaCl, 3.5 KCl, 1.25 NaH_2_PO_4_, 25 NaHCO_3_, 10 glucose, 2 CaCl_2_ and 1 MgSO_4_ and saturated with a 95% O_2_ + 5% CO_2_ gas mixture. For electrophysiological recordings, pipettes were filled with the following whole-cell solution (in mM): K^+^ Methanesulfonate (120), KCl (15), HEPES (10), EGTA (0.1), MgCl_2_, (2), Na_2_PhosphoCreatine (5), Na_2_GTP (0.3), MgATP (2), resulting in a bath resistance of 2–3 MΩ. For coupled recordings of electrical and optical signals, 0.1 mM of Fluo 4 pentapotassium salt was added. Recordings were performed at 34°C. Membrane potential values were corrected for measured junction potential (8 mV) offline. Signals were sampled at 10 kHz and low-pass filtered at 3 kHz with an Axon Multiclamp 700B (Molecular Devices, Sunnyvale, CA, USA). Single or multiple EPSPs were elicited every 15 s (10 pulses at 10 Hz or 40 pulses at 20 Hz; 5–15 V amplitude). Stimuli were delivered with a bipolar tungsten electrode (FHC, Bowdoin, ME, USA), placed at ~200 μm from the soma of the recorded neuron or in the *substantia nigra pars reticulata* (SNr) for recordings of evoked GABA-mediated Inhibitory Post-Synaptic Potentials (IPSPs). During EPSP recordings, neurons were moderately hyperpolarized (~−2/−3 mV) in order to stabilize the membrane potential and hamper the generation of spontaneous or synaptic-driven action potentials (APs). To monitor pharmacological block, Ih-mediated sag potential was elicited by imposing short current pulses (−100 pA, 500 ms) at the end of the sweep. MultiEPSP summation was expressed as the ratio of 10th/1st EPSP amplitude and multiEPSP area was reported as mV × ms. In voltage clamp recordings, access resistance was monitored for the entire duration of the experiment with brief test pulses (−10 mV, 500 ms). Recordings undergoing a drift in access resistance ≥10% were discarded. No whole-cell compensation was used. Ih activation curves were obtained by measuring the amplitude of tail currents at −115 mV following a sequence of 4 s-long test pulses from −45 mV to −125 mV. Electrophysiological and optical traces shown in figures are obtained by averaging five consecutive traces and represent typical observations.

### Microfluorometric Determination of Calcium Responses

Fluorescence signal was collected from a square-shaped window comprising the cell body of the neuron under investigation loaded with 100 μM of the high-affinity, non-ratiometric calcium dye Fluo4 pentapotassium salt (Molecular Probes). Two to three minutes were usually sufficient for complete loading. No differences in major electrophysiological parameters were observed between dye-filled and control neurons. Fluorescence was elicited with a 488 nm LED and collected with a photomultiplier tube (PMT; Cairns Research) with a 10 kHz sampling rate. LED excitation was triggered with the electrophysiological protocol and the PMT signal was acquired and processed as described for voltage signal. SCRs are reported as ΔF/F_0_, where F_0_ signal was the baseline emission of the loaded neuron at rest, and ΔF was defined as F_peak_ − F_0_. Background fluorescence was obtained by measuring the emission of a Fluo 4-free area of the slice and subtracting the obtained value from F_0_. Off-line analysis was performed with Clampfit 10 (Molecular Devices) and Origin 9.1. For the analysis of SCRs kinetics, rise and decay time are intended as the time required to reach 50% of peak. Recordings showing F_0_ decay exceeding 0.5%/s within a single trial, or undergoing irreversible F_0_ rise during consecutive trials were discarded.

### Reagents

Unless otherwise specified, reagents were purchased from Sigma-Aldrich (Saint-Louis, MO, USA). AMPA, NMDA and type 1 metabotropic glutamate receptors (mGluRs) were blocked with, respectively, NBQX, D-APV and CPCOOEt (10 μM, 50 μM, 1 μM; Tocris bioscience, Bristol, UK). GABA_A–B_ receptors were blocked with SR95531 and CGP55845, (10 μM, 1 μM; Tocris). T-type and L-type Voltage-Gated calcium channels (VGCCs) were blocked with mibefradil and isradipine (5 μM each; Tocris). Pharmacological suppression of Ih was obtained with ZD7288 (10 μM; Tocris). Fluo 4 was purchased from Thermo Fisher Scientific (Waltham, MA, USA). GABA_B_ agonist baclofen was used at 1 μM. The K_ATP_ channel blocker glybenclamide was used at 10 μM.

### *In Vivo* Procedures

All *in vivo* procedures were conducted in compliance with the Council Directive of the European Community (2010/63/EU), Decreto Legislativo Italiano 26 (13/03/2014) and approved by the Animal Care Committee of the University of Florence. Male Wistar rats (200–220 g) were purchased from Charles River Laboratories Italia (Lecco, Italy). Animals were housed in humidity- and temperature-controlled room (22–24°C), allowed free access to food (4RF21; Mucedola s.r.l., Milan, Italy) and water, and kept on a 12-h light/dark cycle (lights start at 7:00 AM). Procedures were performed according to a previous report (Provensi et al., 2017), with modifications due to the different brain areas involved. One week after arrival, animals were anesthetized with an intraperitoneal (i.p.) injection of 80 mg/kg zoletil plus 15 mg/kg xylazine and placed on a stereotaxic frame (Stellar, Stoelting Co., Wood Dale, IL, USA). A stainless steel cannula (22 gauge) was implanted bilaterally above the SNpc-VTA boundary (anterior, −5.3 mm; lateral, ± 2 mm; ventral, −6.6 mm from Bregma) and fixed to the skull by using dental cement. Correct cannula placement was verified postmortem. Animals were allowed 3 days to recover from surgery before microinjection procedure. For microinfusions, animals were gently restrained by hand, and an injection needle (30 gauge) was inserted tightly into the guide to 1 mm beyond the end of the guide cannulas. The injection needle was connected to a 1 mL Hamilton microsyringe, and the infusions were performed at a rate of 1 μl/60 s. The injection needle was left in place for an additional 60 s to minimize backflow. It was then withdrawn and placed on the other side, where the procedure was repeated. The drugs used were ZD7288 (5 μg/μl) and Ivabradine (5 μg/μl) dissolved in saline. The volume of the drugs infused was 2 μl per side for four consecutive days. Control groups received equal volumes of sterile saline (0.9%). Correct cannula placement was verified by infusing a 4% (weight/volume) methylene blue solution over 30 s (2 μl). Brains were fixed by transcardiac perfusion 24 h after the last injection with cold physiological saline followed by 4% (volume/volume) paraformaldehyde in 0.1 M phosphate buffer (PB; pH 7.4). Brains were postfixed in the same solution overnight (4°C) and cryoprotected in 30% (weight/volume) sucrose in PB. Cannula placements were considered correct when the spread was 1 mm^3^ or less from the intended infusion sites. Only data from animals confirmed for correct cannula placement were analyzed.

### Behavioral Tests

Twenty-four hours after the last injection, animals were tested for general motor activity with a standard open field test. Animals were positioned in a corner of an open-field arena (*w* = 60 cm; *h* = 30 cm; *d* = 70 cm) and the general motor activity was assessed in 10-min sessions. Animals were monitored with a camera and the covered distance (in cm) measured using a Smart 2.5 software. Animals were then tested for the expression of motor symptoms associated to monolateral DAergic degeneration, by measuring the number of apomorphine-induced rotations. Briefly, animals received an i.p. injection of apomorphine (0.5 mg/kg), then placed in a transparent Perspex cylinder. Animals were video recorded for 30 min. An operator unaware of pharmacological treatments counted the number of rotations.

### Histological Evaluation of DAergic Degeneration

After paraformaldehyde fixation, brains were cut with a cryostate (Leica Microsystems, Buffalo Grove, IL, USA) to obtain 50-μm thick coronal sections of the mesencephalon (~15 sections/brain). Sections were then probed with a mouse monoclonal anti-Tyrosine Hydroxylase (anti-TH) antibody (1:500; Boster Bio, CA, USA) and revealed with an Alexa 488-conjugated secondary antibody (1:500; Abcam, Cambridge, UK). Images were taken with the 10× objective of an epifluorescence microscope (Olympus BX63, Milan, Italy), then digitally reconstructed with the CellSens Dimension software. Quantitative analysis was performed on images of to the ventral half of whole sections according to a previously published method (Gerace et al., 2014). In brief, mean pixel intensity was measured in same-size square regions of interest from the drug-injected and the saline-injected areas of each brain. This value was then normalized to the total intensity of the entire TH-positive area of the drug-injected side and compared to corresponding value of the contralateral, saline-injected side (×5 sections, each brain).

### Data Analysis and Statistics

Pooled data throughout the article are presented as mean ± standard error (SEM) of “n” neurons/animals. Unless otherwise specified, statistical significance was assessed with Student’s *t*-Test for paired measures (Microcal Origin 9.1, Northampton, MA, USA). Graphs and multi-panel figures were generated with Microcal Origin 9.1. Significance at the *p* < 0.05, 0.01, 0.001 and 0.0001 level is indicated with *, **, ***, ****, respectively in figures. Examples of electrical and optical recordings are averages of five traces for each condition and intend to represent typical observations. Temporal summation is expressed as 10th EPSP/1st EPSP ratio. Ih activation curves, normalized I/V plot, fitting and determination of V_1/2_ were generated with Origin 9.1 as described previously (Masi et al., 2013).

## Results

### Properties of Spontaneous SCRs in DAergic Neurons *In Vitro*

**Figure 1:**
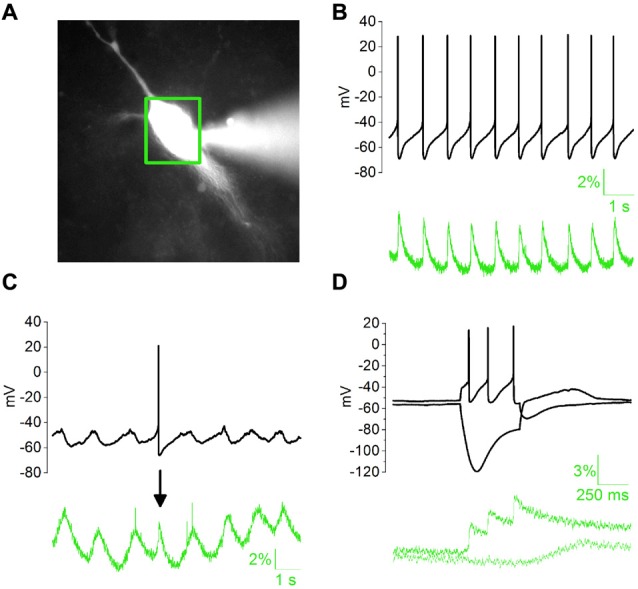
Properties of somatic calcium responses (SCRs) in whole-cell patch clamped dopaminergic (DAergic) neurons in acute midbrain slices. **(A)** Substantia Nigra pars compacta (SNpc) DAergic neuron loaded with 100 μM Fluo 4 pentapotassium salt with a whole-cell pipette (right-hand side) 5 min after patch rupture. Green box indicates the area selected for optical signal collection. **(B)** Typical regular spiking (top) is associated with stereotyped, phase-locked SCRs (bottom). **(C)** Subthreshold oscillatory activity and corresponding slow SCRs. The single action potential (AP)-driven SCR (arrow) in the example trace has faster kinetics but similar amplitude. **(D)** SCRs elicited by somatic square current injection (−100/+100 pA).

Calcium elevations in DAergic neurons may arise from the opening of membrane conductances as well as the mobilization of intracellular calcium pools following activation of inositol 1-4-5 trisphosphate (IP3)-coupled receptors. mGluRs are major mobilizers of calcium from internal stores in DAergic neurons (Cui et al., 2007; Lüscher and Huber, 2010). In addition, external calcium may flow in through calcium-permeable glutamate receptors (Morikawa and Paladini, 2011) or L- and T-type VGCCs (Dufour et al., 2014; Philippart et al., 2016). Initial calcium elevation may, in turn, trigger Calcium-Induced Calcium Release from specific intracellular reservoirs (Morikawa et al., 2000). We tested the sensitivity of our experimental setting by measuring AP-dependent or subthreshold SCRs (Figure 1). During spontaneous discharge of APs (Figure 1B, black trace), SCRs appeared as AP-locked positive spikes (green trace, 2.967 ± 0.59%), with distinct rise and decay kinetics (rise, 97.99 ± 8.64 ms; decay, 153.88 ± 11.08 ms, *N* = 7). During spontaneous subthreshold oscillations (Figure 1C), SCRs had comparable amplitude (2.78 ± 0.13%), but slower kinetics (rise, 333.29 ± 19.37 ms; decay, 438.52 ± 16.43 ms, *N* = 5) compared to the previous. In response to pipette-stimulated AP bursts, fluorescence signal showed sustained elevation, due to transient SCR summation. In contrast, somatic hyperpolarization elicited a modest downward deflection of calcium signal followed by a positive rebound at the end of negative current step (Figure 1D).

### Voltage-Dependent Component of Evoked EPSP-Induced SCRs

**Figure 2:**
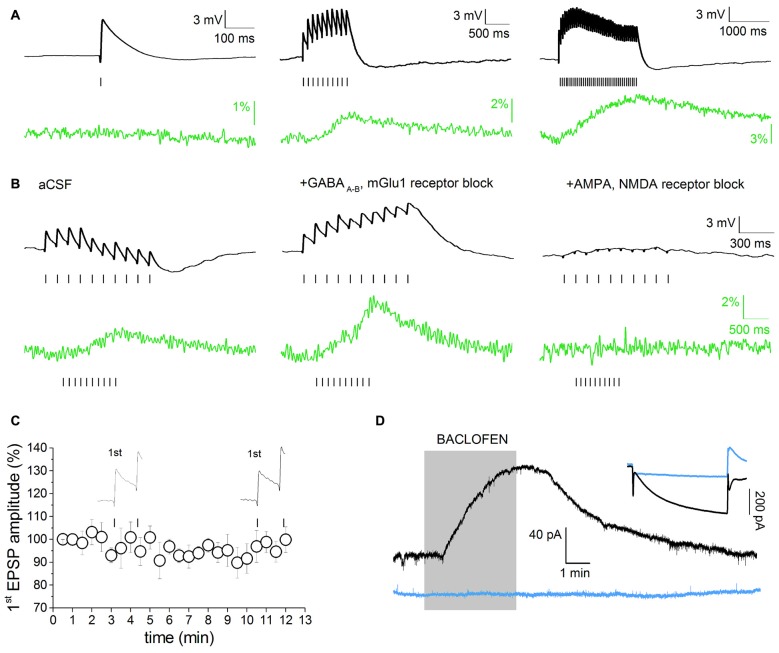
Voltage-dependent component of evoked excitatory post-synaptic potential (EPSP)-induced SCRs. **(A)** Single or multiple evoked EPSPs (black), in absence of pharmacological isolation, and relative Fluo 4 traces (green). Traces are baseline-adjusted to facilitate comparison. Single EPSPs are not able to elicit detectable SCRs (left). A short EPSP train (10 Hz/1 s) is able to elicit a measurable SCR (middle). Twenty hertz/two seconds stimulation protocols elicit prolonged, slow-decaying, but not saturating, SCRs (right). **(B)** multiEPSP and relative SCRs recorded before (left) and after superfusion with GABA_A_, GABA_B_ and metabotropic glutamate receptors 1 (mGlu1) receptor antagonists (SR95531, 10 μM; CGP55485, 1 μM and CPCCOEt, 1 μM; middle). Subsequent administration of AMPA and NMDA receptor antagonists (NBQX, 10 μM; D-APV, 50 μM) fully abolishes the SCR (right). Note that voltage and optical traces have different time scales. **(C)** The amplitude of first EPSP does not change during 10 min of synaptic stimulation with the 10 Hz/1 s protocol in presence of GABA_A–B_ and mGlu1 antagonists. **(D)** Characterization of Ih-positive, putative DAergic neurons in the ventral tegmental area (VTA) based on the electrophysiological response to bath application of the GABA_B_ agonist baclofen (1 μM). The inset shows the whole-cell current in response to a −95 mV step. Eight/nine neurons showing I ≥ 200 pA responded to baclofen. Ih-negative, presumed non-DAergic neurons do not respond to baclofen (blue traces).

Simultaneous recordings of evoked synaptic activity and SCRs were obtained in DAergic neurons of the SNpc or the VTA in acute horizontal slices prepared from P20 to P30 Wistar rats (Figure 2). The study was restricted to neurons with typical DAergic morphology and physiological properties (large, polygonal or fusiform cell body, broad AP, Ih ≥ 200 pA). Morphological and electrophysiological properties are good predictors of DAergic phenotype in the SNpc. In the VTA, DAergic neurons were selected on the basis of the specified electrophysiological phenotype and their position relative to the medial terminal nucleus of the accessory optic tract (MT; Neuhoff et al., 2002; Margolis et al., 2006). In addition, because in the VTA standard DAergic markers are less specific (Margolis et al., 2010), a separate group of DAergic-like VTA neurons were challenged with the GABA_B_ agonist baclofen (1 μM). The activation of an outward potassium current following baclofen administration is a good predictor of DAergic phenotype (Margolis et al., 2012). Eight out of nine neurons showing a presumed DAergic phenotype (morphology, Ih ≥ 200 pA) responded to baclofen (Figure 2D), thus confirming the reliability of the selection criteria described above. It has been demonstrated that HCN channels reduce synaptic excitability in many neuronal types by dampening the amplitude and duration of EPSPs (He et al., 2014). Consistently, Ih inactivation enhances dendritic calcium spikes in CA1 pyramidal neurons due to increased EPSP temporal summation and T-/N-Type VGCC recruitment (Tsay et al., 2007). However, it has not been tested whether this phenomenon occurs in midbrain DAergic neurons, or whether it has any significance in DAergic pathologies. To address this question, we optimized a stimulation paradigm producing subthreshold EPSPs and simultaneously measured the changes in emission intensity of the high-sensitive, non-ratiometric dye Fluo 4. Stimulation conditions such as frequency, amplitude and duration of stimuli, and baseline potential were adjusted as not to determine plasticity of synaptic responses or irreversible calcium accumulation (Jones and Bonci, 2005; Kauer and Malenka, 2007; Harnett et al., 2009). Preliminary studies of EPSP-driven calcium signal properties were carried out in SNpc DAergic neurons. Figure 2A shows representative single and multiple EPSPs and associated calcium signals elicited with protocols of increasing duration and frequency, designed to assess the input-output properties of our synaptic stimulation protocol. Single EPSPs (peak amplitude: 4.43 ± 0.53 mV, baseline potential: −62.75 ± 1.06 mV, *n* = 9) failed to elicit measurable SCRs in all neurons tested (Figure 2A, left). Ten stimuli at 10 Hz (10 Hz/1 s) produced multiple subthreshold EPSPs (area, 7836 ± 996 mV × ms, *n* = 23) and corresponding SCRs (2.33 ± 0.53%, *n* = 23; Figure 2A, middle). This response was largely below maximal level, as a train with twice the stimulation intensity (20 Hz/2 s) was still able to produce non-saturating SCRs (6.53 ± 1.43%, *n* = 5; Figure 2A, right), although this protocol occasionally caused progressive F_0_ rise, likely due to irreversible calcium accumulation. Therefore, the 10 Hz/1 s stimulation protocol was used for all experiments and referred to as “multiEPSP” henceforth throughout text and legends. In SNpc DAergic neurons, synaptic activation of type-1 metabotropic glutamate receptors (mGlu1) leads to the release of calcium from intracellular stores with an IP3-mediated, Ryanodine-sensitive mechanism (Fiorillo and Williams, 1998; Morikawa et al., 2003). We pharmacologically eliminated this major voltage-independent calcium response by using the mGlu1 selective antagonist CPCOOEt (10 μM). GABA_A–B_ receptor transmission was also blocked with SR95531 (10 μM) and CGP55845 (1 μM) in order to isolate the AMPA/NMDA receptor-mediated component of the multiEPSP and the corresponding SCR. Figure 2B shows the effect of GABA_A–B_ and mGlu1 receptor blockers (“control solution” henceforth) on multiEPSP and SCR. Note that abrogation of the slow mGlu1 receptor-dependent hyperpolarization (Fiorillo and Williams, 2000) results in the elevation of multiEPSP area and SCR peak as shown in an example recording (Figure 2B, middle). AMPA/NMDA receptor blockers fully abolished both multiEPSP and SCR, suggesting that calcium transients are sustained by activation of NMDA receptors and VGCCs in these experimental conditions (Figure 2B, right). We verified that the 10 Hz/1 s protocol did not elicit forms of synaptic plasticity by monitoring the amplitude of the first EPSP while stimulating multiEPSP for 10 min in the control solution (Figure 2C). Figure 2D shows the response to baclofen of 8/9 Ih-positive VTA neurons (black traces). Blue traces refer to an Ih-negative VTA neuron which did not respond to baclofen.

### MultiEPSP-Dependent SCRs in SNpc DAergic Neurons Are Mainly Mediated by L-Type Calcium Channels

**Figure 3:**
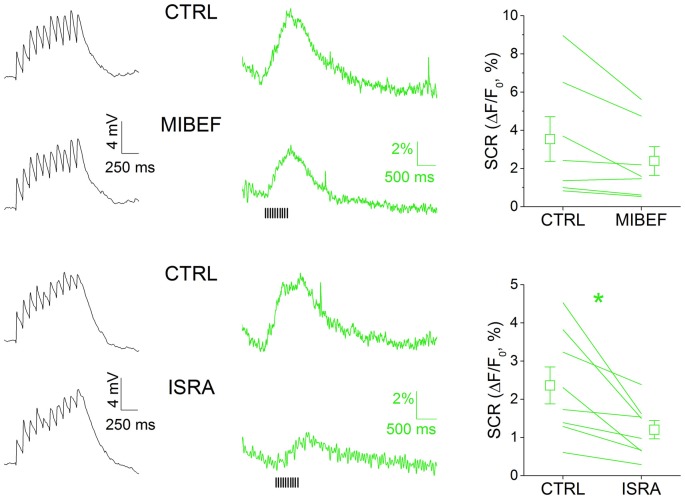
MultiEPSP-dependent SCRs in SNpc DAergic neurons are mainly mediated by L-type calcium channels. SCRs were tested with the T- and L-type calcium channels’ antagonists mibefradil and isradipine (5 μM, each). Both drugs are able to reduce SCRs, but only in the case of isradipine the effect achieved statistical significance. No effect of T- and L-type calcium channels block was seen on multiEPSP (left, black traces).

We sought to characterize the nature of the calcium rise triggered by multiEPSP in SNpc DAergic neurons with a pharmacological approach (Figure 3). We did not systematically test the contribution of NMDA receptor activation on SCR because NMDA receptor antagonism negatively affects EPSP kinetics (not shown) and because it was reported that NMDA receptor-mediated calcium transients are modest in the somatodendritic compartment of SNpc DAergic neurons (Jang et al., 2011). In these neurons, members of the CaV 1 and 2 families have previously been identified as the VGCC pore-forming subunits with activation windows in the subthreshold domain (Chan et al., 2007; Dufour et al., 2014; Poetschke et al., 2015; Philippart et al., 2016), thus most likely involved in calcium inflow associated to subthreshold depolarization episodes such as those triggered by our stimulation protocol. We tested the involvement of T- and L-type VGCCs in multiEPSP-triggered SCRs in SNpc DAergic neurons by using, respectively, mibefradil (5 μM) and isradipine (5 μM). Mibefradil reduced SCR, but the effect did not reach statistical significance (CTRL, 3.54 ± 1.17%; mibefradil, 2.39 ± 0.75%, *n* = 7, *p* = 0.177). Isradipine, in contrast, caused a near 50%, significant reduction of SCR (CTRL, 2.36 ± 0.48%; isradipine, 1.20 ± 0.24%; *n* = 8, *p* = 0.014). Of note, no alteration of the amplitude or kinetics of the multiEPSP was observed with either blocker (black traces), suggesting a post-synaptic location of drug targets. These results are consistent with the previously mentioned evidence demonstrating the expression of CaV 1.2 and 1.3 L-type calcium channels in the somatodendritic compartment of SNpc DAergic neurons, and their role at subthreshold potentials.

### Ih Block Potentiates MultiEPSP and MultiEPSP-Dependent SCRs in SNpc DAergic Neurons

**Figure 4:**
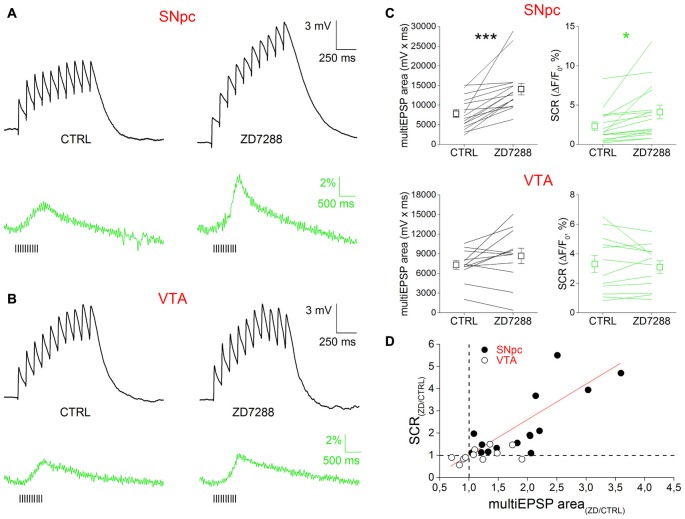
Ih block potentiates multiEPSP and multiEPSP-dependent SCR in SNpc DAergic neurons. **(A)** Effect of Ih block in SNpc DAergic neurons. Example of multiEPSP in presence of GABA_A–B_ and mGluR1 antagonists (“CTRL”) and after application of the specific Ih blocker ZD7288 (10 μM, 10 min). The expected membrane hyperpolarization (−5.11 ± 0.77 mV) and multiEPSP area increase (top) is accompanied by potentiation of the SCR (bottom). Voltage traces are baseline-adjusted to facilitate comparison. **(B)** No hyperpolarization (−0.85 ± 1.07 mV), change in multiEPSP area or SCR peak following Ih blockade in VTA DAergic neurons. **(C)** Group data and statistical analysis relative to ZD7288-induced changes in multiEPSP and SCR in the two areas. **(D)** SCR and multiEPSP variations caused by ZD7288 have a positive linear correlation.

The ability of Ih to affect multiEPSP and multiEPSP-driven SCRs was tested using the specific blocker ZD7288 (10 μM, 10 min) in bath application (Figure 3). Prior to drug application, multiEPSP and corresponding SCRs were recorded for 5 min in the control solution to achieve a stable baseline. In SNpc DAergic neurons (Figure 4A), perfusion with ZD7288 significantly hyperpolarized the neuron (−5.117 ± 0.777 mV; *n* = 23, *p* = 1.75 × 10^−5^) while increasing multiEPSP temporal summation (CTRL, 2.165 ± 0.374; ZD7288, 3.221 ± 0.688; *n* = 23, *p* = 0.0128) and multiEPSP area (CTRL, 7836 ± 996; ZD7288, 14,087 ± 1473; *n* = 23, *p* = 1.63 × 10^−4^). Of note, multiEPSP-driven SCR increased twofold following HCN block (CTRL, 2.33 ± 0.53%; ZD7288, 4.68 ± 1.32%; *n* = 23, *p* = 0.044). In contrast, ZD7288 application did not alter membrane potential (−0.854 ± 1.069 mV, *n* = 12, *p* = 0.445), multiEPSP summation (CTRL, 2.819 ± 0.419; ZD7288, 3.648 ± 0.762; *n* = 12; *p* = 0.316), and area (CTRL, 7317 ± 675; ZD7288, 8683 ± 1180; *n* = 12; *p* = 0.122), or SCR (CTRL, 3.81 ± 0.85%; ZD7288, 2.94 ± 0.37%, *n* = 12, *p* = 0.184) in the VTA. Comparatively, there was no significant difference in mean multiEPSP summation, multiEPSP area or SCR amplitude between SNpc and VTA DAergic neurons in control conditions (*p* = 0.256, *p* = 0.69 and *p* = 0.16, respectively; *n* = 23 and 12, two sample *t* Test). In presence of ZD7288, the two populations diverged significantly in terms of magnitude of multiEPSP area (SNpc, +110.65 ± 25.68%; VTA, +12.36 ± 13.47%, *n* = 23 and 12, *p* = 0.0026, two sample *t* Test) and SCR (SNpc, +128.25 ± 34.73; VTA, −3.87 ± 9.62%, *n* = 23 and 12, *p* = 0.0035, two sample *t* Test), but not of multiEPSP summation (SNpc, 3.222 ± 0.688; VTA, 3.647 ± 0.763, *n* = 23 and 12, *p* = 0.682, two sample *t* Test). Globally, SCR increase was related to multiEPSP area increase by a linear function (*y* = *a* + *bx*; *a* = 0.307; *b* = 0.835; *r* = 0.641, *p* = 2.35 × 10^−4^), indicating the strong voltage-dependent nature of SCRs. Overall, these results indicate that Ih inhibition potentiates synaptic excitability and depolarization-dependent somatic calcium entry. Quantitatively, effect magnitude reaches statistical significance only in the SNpc, consistent with the previously reported stronger influence of Ih over synaptic excitability in this subset (Masi et al., 2015).

### Ih Suppression Depresses GABA_A_ Responses in SNpc DAergic Neurons

**Figure 5:**
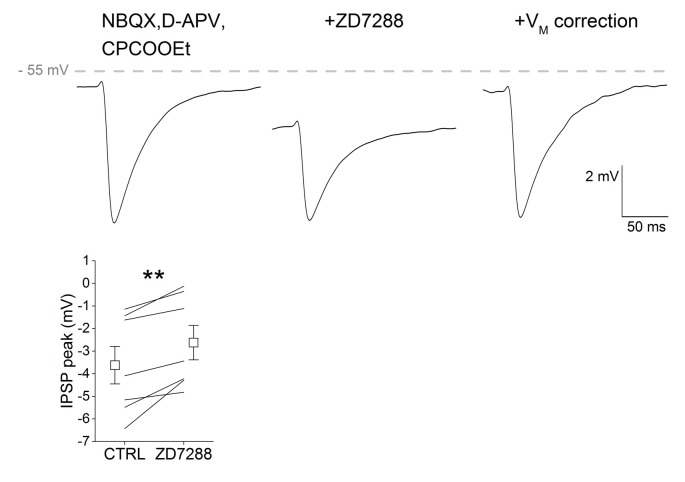
Ih suppression depresses GABA_A_ responses in SNpc DAergic neurons. Example of a single, pharmacologically isolated inhibitory post-synaptic potential (IPSP) obtained with extracellular stimulation of the Substantia nigra pars reticulata (SNr) and recorded from a SNpc DAergic neuron (top). ZD7288 causes membrane hyperpolarization accompanied by a significant reduction of peak amplitude (middle). IPSP amplitude recovers control amplitude when cell membrane is manually held at original potential (right). Bottom, group data and statistical analysis relative to ZD7288-induced change in IPSP amplitude.

It has been shown that Ih antagonism reduces the amplitude of GABA_A_-mediated IPSPs in CA1 pyramidal neurons by hyperpolarizing the membrane potential and thus reducing GABA_A_ receptor driving force (Pavlov et al., 2011). We tested whether this was also the case in SNpc DAergic neurons by electrically stimulating GABAergic neurons in the SNr and recording single evoked IPSPs from SNpc DAergic neurons in presence of glutamatergic synaptic blockers (Figure 5). We found that IPSP amplitude was significantly reduced following bath application of ZD7288 (CTRL, −3.622 ± 0.83; ZD7288, −2.62 ± 0.76, *n* = 7, *p* = 0.005). The effect of Ih block on IPSP amplitude depends on the negative shift in membrane potential, as indicated by the recovery of IPSP amplitude obtained by offsetting the ZD7288-induced hyperpolarization. These results indicate that Ih block increases synaptic excitability with a dual mechanism, that is by increasing the response to excitatory inputs and by reducing that to inhibitory inputs.

### Low Intracellular ATP Causes a Negative Shift in Ih Activation Curve

**Figure 6:**
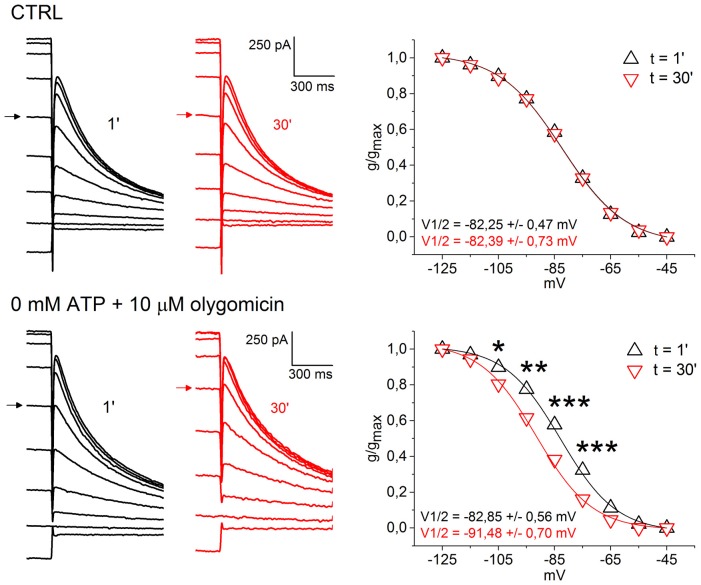
Low intracellular ATP causes a negative shift in Ih activation curve. Top, example of voltage clamp recordings at 1 min (black) and 30 min (red) after patch rupture with control pipette solution. Respective Ih activation curves show perfect overlap (right). Bottom, same experiment, but using a pipette solution with 0 mM ATP and 10 μM oligomycin. Ih activation curve at 30 min is left-shifted by ~9 mV (right). Arrows indicate current traces obtained at the −85 mV test potential to highlight the shift in voltage dependence.

Disruption of mitochondrial homeostasis is increasingly seen as a fundamental pathogenic process involved in both familial and sporadic forms of PD (Schapira and Gegg, 2011). Animal models generated by intoxication with mitochondrial poisons, such as MPP+, or by introduction of DAergic-targeted mitochondrial defect, as in the case of the MitoPark model, are characterized SNpc-specific DAergic degeneration (Ekstrand and Galter, 2009; Blesa and Przedborski, 2014). Both models have been associated to Ih loss of function, therefore we asked whether HCN channels in SNpc DAergic neurons, *in vitro*, respond to block of mitochondrial ATP synthesis and low ATP levels. To this aim, we recorded Ih activation curves using a whole-cell pipette solution containing 0 mM ATP and 10 μM oligomycin, a selective blocker of mitochondrial F_0_-F_1_ ATP synthase (Figure 6). Olygomicin causes the block of mitochondrial, but not glycolytic, ATP synthesis, leaving the transmembrane mitochondrial potential unaffected (Nicholls and Budd, 2000; Schuchmann et al., 2000). As a control, we used a standard K methanesulfonate-based solution containing 2 mM ATP. Ih activation curves were recorded immediately after patch rupture and 30 min later. Figure 6 shows example traces (left) and activation curves (right) obtained with control (top) and 0 ATP/oligomycin (bottom) pipette solution. No changes in Ih activation curve was observed with control pipette solution (V_1/2_ *t*_0_ = −82.25 ± 0.47 mV; V_1/2_ *t*_30_ = −82.39 ± 0.73 mV; *n* = 5, *p* = 0.269). In contrast, low ATP caused a highly-significant, negative shift in Ih activation curve (*t*_0_, V_1/2_ = −82.85 ± 0.56 mV; *t*_30_, V_1/2_ = −91.48 ± 0.70 mV; *n* = 7, *p* = 0.000589). Importantly, the effect occurred in the absence of detectable changes in holding current and membrane resistance. In one case, low ATP solution caused the appearance of an outward current, which was fully reverted by application of 10 μM glybenclamide, a selective K_ATP_ channel blocker (not shown). The magnitude of the shift in voltage dependence was consistent with the effect exerted by changes in cAMP levels on HCN2 and HCN4 (Biel et al., 2009), the two main Ih pore-forming subunits in SNpc DAergic neurons (Franz et al., 2000; Dufour et al., 2014), suggesting that impaired cAMP synthesis is the mechanism of action involved here.

### Local Pharmacological Ih Suppression Causes Preferential SNpc DAergic Degeneration and Hemiparkinsonism

**Figure 7:**
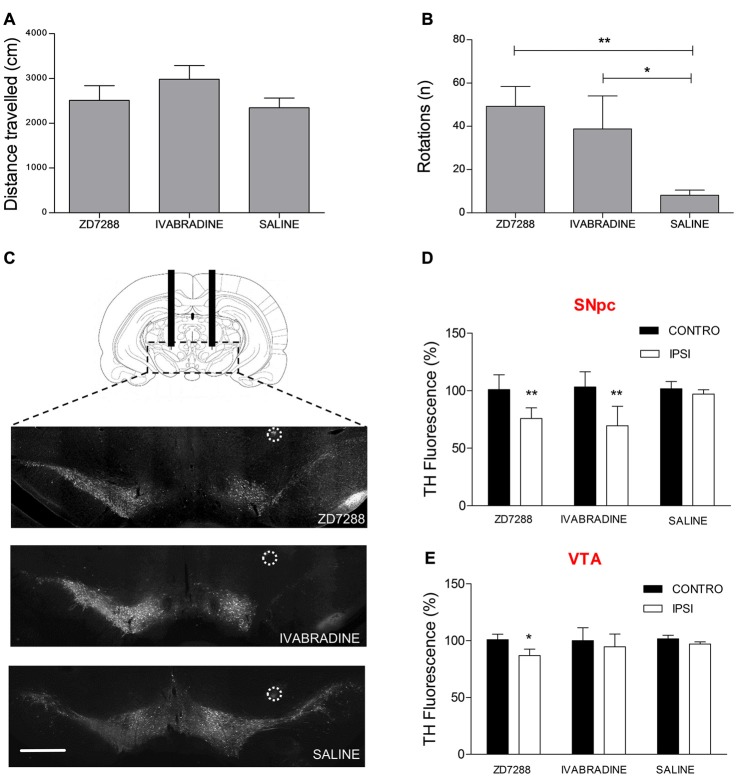
Local pharmacological Ih suppression causes preferential SNpc DAergic degeneration and hemiparkinsonism. Spontaneous locomotion **(A)** and apomorphine-induced rotations **(B)** in rats after repeated monolateral infusions of ZD7288 and Ivabradine (1/day × 4 days). **(C)** Representative microphotographs showing tyrosine hydroxylase (TH) immunofluorescent labeling on midbrain coronal sections. Circles indicate histologically-confirmed injection sites (scale bar = 1 mm). **(D,E)** Densitometric analysis of ZD7288- and Ivabradine-induced DAergic degeneration in the SNpc and VTA.

We tested the hypothesis that Ih suppression participates in the pathogenic cascade eventually leading to preferential degeneration of nigrostriatal DAergic neurons. To this aim, we performed intracerebral injections of two distinct Ih blockers following stereotaxic coordinates in adult rats. Injection site was 1 mm above the SNpc-VTA boundary. ZD7288 and Ivabradine (5 μg/μl, 2 μl each) were injected once a day for four consecutive days on one hemisphere, while the contralateral hemisphere was injected with an equal volume of saline. On the fifth day, animals were first tested for spontaneous motility then for the expression of apomorphine-induced rotation behavior, a typical sign of monolateral nigrostriatal degeneration (Hudson et al., 1994). As shown in Figure 7A, ZD7288- or Ivabradine-treated animals did not show changes in spontaneous locomotion compared to controls (ZD7288, 2511 ± 328 cm, *n* = 8; Ivabradine 2982 ± 303 cm, *n* = 5; saline 2345 ± 216 cm, *n* = 8; one way ANOVA + Newman-Keuls *post hoc* test, *F*_(2, 20)_ = 1.098, *p* = 0.354, for differences among treatments). In contrast, both treatments significantly increased the number of apomorphine-induced rotations (ZD7288, 49 ± 9 turns in 30 min, *n* = 8, Ivabradine, 39 ± 15, *n* = 4, saline, 8 ± 2, *n* = 8, *p* = 0.004, one way ANOVA + Newman-Keuls *post hoc* test, *F*_(2,19)_ = 7.671, *p* < 0.004 for differences among treatments; Figure 7B). Afterwards, DAergic degeneration was assessed by performing TH immunostaining in coronal sections from the mesencephalon of treated and control animals. As clearly shown by the representative microphotographs (Figure 7C), the intensity of TH staining is significantly decreased in the SNpc of the drug-injected side compared to saline-injected side (ZD7288, 75.86 ± 4%, *n* = 8, *p* = 0.001 vs. control; Ivabradine, 69.51 ± 8.9%, *n* = 6, *p* = 0.001 vs. control; Figure 7D). In contrast, the effect of ZD7288 was much smaller in the VTA, while Ivabradine failed to achieve statistical significance (ZD7288, 86.91 ± 4%, *n* = 8, *p* = 0.01 vs. control; Ivabradine, 94.78 ± 7.9%, *n* = 6, *p* = 0.7 vs. control; Figure 7E). Overall, these results suggest that Ih suppression is sufficient to cause degeneration of DAergic neurons. Furthermore, degeneration is not homogeneous, as SNpc DAergic neurons are strikingly more sensitive to the effects of Ih suppression compared to VTA DAergic neurons.

## Discussion

**Figure 8:**
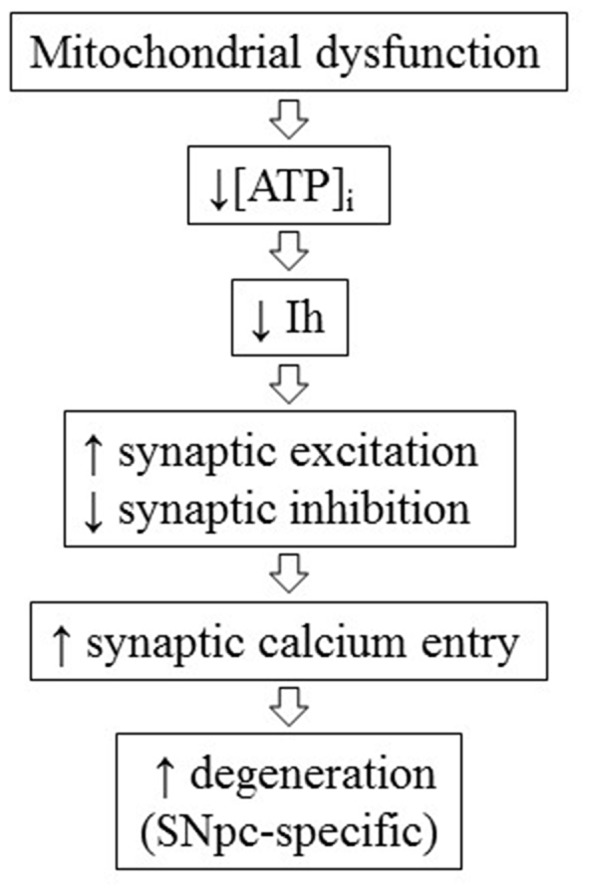
Conceptual scheme of the hypothetical pathogenic cascade linking mitochondrial dysfunction, Ih loss of function and SNpc-specific neurodegeneration during the progression of Parkinson’s disease (PD).

In the first part of the present work, by using whole-cell patch clamp electrophysiology and microfluorometric calcium measurements in rat brain slices, we demonstrate that Ih not only determines synaptic excitability in DAergic neurons as we previously showed (Masi et al., 2015), but also indirectly regulates synaptic-driven, voltage-dependent calcium entry. This function is area-specific, as Ih pharmacological blockade causes significant enhancement of multiEPSP and associated SCR in the SNpc, while neither parameter is significantly affected in the VTA. This is consistent with the differential control exerted by Ih over synaptic excitability between the two areas in TH-GFP mice (Masi et al., 2015). Of note, functional Ih diversity between SNpc and VTA was quantitative in TH-GFP mice, while it appears to be qualitative in rats, a discrepancy which presumably results from the species difference. We pharmacologically characterized the source of calcium recruited by multiEPSP in SNpc DAergic neurons with the L- and T-type VGCCs blockers isradipine and mibefradil. Only isradipine was effective in causing a significant reduction of SCRs. This finding does not allow discrimination between CaV 1.2 and 1.3, as the two pore-forming subunits show comparable affinity for the drug (Zamponi et al., 2015), while the non-significant effect produced by mibefradil indicates a limited contribution of T-type calcium channels to SCRs, in agreement with the relatively hyperpolarized activation window of these channels. The contribution of an NMDA receptor-dependent calcium component to SCRs, although possible in our experiments, was not tested because NDMA receptor blockers alter EPSP kinetics, thus confounding the effect of Ih blockade on dendritic integration. Moreover, it has been shown that NMDA receptor-dependent calcium inflow is relatively modest at proximal dendritic locations, while VGCC-dependent calcium inflow is relatively large, suggesting a limited contribution of the first to SCRs, at least in quantitative terms (Jang et al., 2011). In agreement, another study has reported strong expression of CaV 1.2, 1.3 and CaV 3.1, 3.2, 3.3 pore-forming subunits in the somatodendritic compartment of SNpc DAergic neurons in rats (Dufour et al., 2014). Our results imply that one physiological function of Ih is to limit synaptic-dependent calcium entry. Ih suppression enhances AMPA/NMDA receptor-mediated response and blunts GABA_A_ receptor-mediated response, thus increasing synaptic excitability in a dual manner. In this work, we also show that Ih is functionally regulated by intracellular ATP concentration. Low intracellular ATP, obtained by using a pipette solution containing 0 mM ATP and 10 μM of the F_0_-F_1_ ATP synthase blocker oligomycin, causes a ~−10 mV negative shift in Ih activation curve. This is compatible with the reported cAMP sensitivity of HCN2 and HCN4 (Biel et al., 2009), the two isoforms sustaining Ih in SNpc DAergic neurons (Franz et al., 2000; Dufour et al., 2014). As there is no evidence for a direct modulation of HCN channels by ATP, the observed shift in the voltage dependence is likely due to reduced cAMP synthesis. Importantly, this experiment suggests a mechanistic link between ATP levels, Ih-dependent synaptic excitability and calcium dynamics. In this regard, there is increasing evidence linking mitochondrial damage to Ih function. Ih is suppressed by the mitochondrial toxin MPP+ *in vitro* (Masi et al., 2013) and lamotrigine, a commercial anticonvulsant agent reported to activate Ih (Poolos et al., 2002; Friedman et al., 2014), is neuroprotective in MPTP-induced DAergic degeneration models (Archer and Fredriksson, 2000; Lagrue et al., 2007). Furthermore, Ih current density is diminished in SNpc DAergic neurons of MitoPark mice at 6 weeks of age, well before the appearance of neurodegeneration (Good et al., 2011). This finding is consistent with the evidence, presented here, that Ih is functionally coupled to ATP levels. Of note, another more recent work has reported a reduction in Ih current density in surviving DAergic neurons in a rat model showing spontaneous α-synuclein accumulation, suggesting that the pathogenic relevance of Ih loss of function may not be limited to mitochondrial models (Guatteo et al., 2017). Here, we show that local administration of Ih blockers leads to DAergic degeneration in adult rats. Like in many established PD models, DAergic degeneration is most severe in the SNpc, while the VTA appears substantially spared. In view of PD pathogenesis, Ih loss of function may possibly be regarded as an acquired alteration, caused by disruption of mitochondrial metabolism, affecting specifically, or to a larger extent, DAergic neurons in the SNpc, where Ih is critical in the regulation of synaptic excitability. SNpc DAergic neurons receive a tonic excitatory input from the Subthalamic nucleus (STN), which is disinhibited during PD progression as a consequence of striatal dopamine loss. Experimental ablation of this input protects SNpc DAergic neurons from MPTP and 6-OHDA-induced degeneration (Piallat et al., 1996; Wallace et al., 2007), suggesting the involvement of an excitotoxic pathogenic mechanism in these models. Therefore, it is conceivable that STN disinhibition may add to the excitation/inhibition unbalance caused by Ih loss of function and eventually determine a SNpc-specific pathogenic pathway involving L-type VGCCs activation, as schematized in Figure 8. CaV 1.3-mediated dendritic calcium oscillations, characterizing rhythmic activity of SNpc DAergic neurons, have previously been suggested to determine the vulnerable phenotype of these neurons (Guzman et al., 2009; Ilijic et al., 2011; Surmeier and Schumacker, 2013). In agreement, clinical studies and meta-analyses indicate that users of brain-permeable L-type VGCC blockers have a 30% reduced risk of developing PD (Ritz et al., 2010; Lang et al., 2015). Our results indicate that L-type calcium channels are activated by normal subthreshold synaptic activity and become hyperactivated in SNpc DAergic neurons following pharmacological Ih suppression. *In vivo*, Ih loss of function may result from mitochondrial dysfunction, a key disease mechanism at the basis of extensively studied PD animal models, which is gaining increasing attention in the human pathology too. To conclude, the present research supports the hypothesis that Ih loss of function represents a bona fide pathogenic mechanism which, possibly in concert with SNpc-specific connectivity, may determine differential DAergic vulnerability during disease progression in relevant animal models and in human PD.

## Author Contributions

CC, AC and GP performed experiments. GM supervised research and revised the manuscript. AM designed research, performed experiments, analyzed data and wrote the manuscript. CC and AC contributed equally.

## Conflict of Interest Statement

The authors declare that the research was conducted in the absence of any commercial or financial relationships that could be construed as a potential conflict of interest.
